# CircFN1 promotes acute myeloid leukemia cell proliferation and invasion but refrains apoptosis via miR‐1294/ARHGEF10L axis

**DOI:** 10.1002/kjm2.12801

**Published:** 2024-01-05

**Authors:** Sheng Wang, Bang‐Shuo Zhang, Yi Yang, Lin‐Lin Fu

**Affiliations:** ^1^ Department of Hematology Three Gorges Hospital Affiliated to Chongqing University Chongqing City China

**Keywords:** acute myeloid leukemia, ARHGEF10L, cell proliferation, circFN1, miR‐1294

## Abstract

Previous studies have proved circFN1 is highly expressed in acute myeloid leukemia (AML) patients and AML cell lines. This study aims to investigate the impact of circFN1 on AML and its mechanism. Via real‐time quantitative PCR to detect circFN1, miR‐1294, ARHGEF10L expressions in clinical plasma samples and AML cell lines, AML cells were cultured in vitro and transfected with si‐circFN1, pcDNA3.1‐circFN1, and si‐ARHGEF10L, respectively, or co‐transfected pcDNA3.1‐circFN1 + miR‐1294 mimic and pcDNA3.1‐circFN1 + si‐ARHGEF10L. Using dual luciferase reporter experiment to detect the relationship between circFN1 and miR‐1294, as well as miR‐1294 and ARHGEF10L. CCK‐8 was used to detect cell proliferation, Transwell to cell invasion, TUNEL staining and flow cytometry to detect cell apoptosis, RT‐qPCR to circFN1 RNA, miR‐1294, and ARHGEF10L expression levels in HL‐60 cells, and western blot to ARHGEF10L protein expression level in HL‐60 cells. We found highly expressed circFN1 and ARHGEF10L, as well as low‐expressed miR‐1294 in AML patients and AML cell lines. In contrast to si‐NC group, si‐circFN1 group could signally inhibit HL‐60 cell proliferation and migration, but promote cell apoptosis; compared with mimic NC group, miR‐1294 mimic group could visually inhibit HL‐60 cell proliferation and migration, but promote cell apoptosis. miR‐1294 was the target of circFN1, and ARHGEF10L was the target of miR‐1294. Over‐expressing miR‐1294 or silencing ARHGEF10L could signally inhibit circFN1 promoting HL‐60 cell proliferation and migration and repressing cell apoptosis. circFN1 promotes proliferation and invasion of AML cell and represses cell apoptosis via regulating miR‐1294/ARHGEF10L axis, which provides new insight for molecular targeted‐treatment for AML.

## 1 INTRODUCTION

Acute myeloid leukemia (AML) is a heterogeneous leukemia occurring in the myeloid, erythroid, megakaryocyte, and monocyte precursors. AML is defined as a devastating and rapidly developing disease, features rapid immature myeloid progenitor cell proliferation, which was blocked at various stages of myeloid differentiation. At the molecular level, AML is a heterogeneous genetic disease resulting from different mutations and translocations. These genetic changes usually result in transcription factor imbalance, which is essential for controlling the expansion and lineage determination of myeloid progenitor cells.^1^ Clonal chromosome rearrangement is present in approximately 55% of AML patients, including *t* (8; 21), *t* (15; 17), and *t* (16; 16), resulting in fusion genes RUNX1/RUNX1T1, PML‐RARα, and CBFB‐MyH11, respectively.^2^ Some of these mutations are clinically associated with diagnostic or prognostic markers and potential therapeutic targets.^3^ According to the French‐American‐British system, there are 8 types of AML (M0–M7), with specific morphological characteristics and differentiation stages.^4^ The more recent World Health Organization classification is based on a combination of clinical symptoms, cell morphology, immunophenotype, and genetic abnormalities.^5^ In most cases, the disease involves the bone marrow, and malignant cells or extramedullary immersion can also be detected in peripheral blood. APL is a variant of AML that has specific genetic abnormalities of leukemic cells and severe clotting disorders, and its treatment differs from other types of AMLP1. For the vast majority of patients, AML mainly affects the bone marrow, and at least 20% of the nucleated bone marrow cells are immature or undifferentiated leukemic mother cells. AML can be divided into neotype, secondary and AML according to its cause.^6^ The incidence of AML increases with age, and because leukemia stem cells cannot be eradicated, resulting in a high rate of AML recurrence, the treatment outcome and prognosis of AML remain poor. Recently, the treatment of AML has taken on a new dimension due to the emergence of many new therapies such as targeted therapies.^7^ But, most AML patients will relapse within 3 years after diagnosis.^8^ Therefore, the search for new anti‐cancer target drugs is essential for AML targeted therapy, recurrence, metastasis, and prognosis improvement.

Circular RNA (circRNA), a conserved single‐stranded RNA molecule, stems from exon or intron sequence through reverse shear by precursor mRNA.^9^ Unlike standard linear RNA, circRNA forms a covalently closed, continuous stable loop, with no 5′ end cap and 3′ end poly (A) tail, therefore, it is resistant to exonuclease digestion.^10^, ^11^ Most circRNAs, abundant and conserved in different species, has specific expression in tissues or developmental stages.^9^ Studies have revealed that circRNAs act in various cancers and can be used as better predictive biomarkers as well as a therapeutic targets for cancer treatment.^12^, ^13^, ^14^, ^15^ CircRNA is out of control in cancer and leukemia,^16^, ^17^, ^18^, ^19^ and available to promote disease pathogenesis through affecting the characteristics of cancer.^19^ CircFN1 is highly expressed in gastric cancer cells with the impact of inhibiting cell apoptosis and elevating cell resistance.^20^ CircFN1 has the potential to be used as a drug target for treating AML. Most circRNAs can be used as miRNA sponges to regulate miRNA function through inhibiting the impact of ceRNA.^21^ miR‐1294 inhibits cancer cell growth in various cancers.^22^, ^23^, ^24^ However, the role of miR‐1294 in AML is still not clear. Starbase database analysis found that CircFN1 has a direct binding site with miR‐1294. The downstream mRNA of miR‐1294 was predicted through online prediction software microT, PicTar, PITA, and RNA22. Using Venn diagram and GEPIA online database analysis, it was found that ARHGEF10L and SULF2 were highly expressed in AML. ARHGEF10L is available to promote liver cancer occurrence,^25^ but the mechanism and role of ARHGEF10L in AML have not been reported yet.

Our research aims to demonstrate the mechanism and role of circFN1 in AML, which will expand them in AML, with a view to finding new targets for treating AML from a genetic perspective.

## 2 MATERIALS AND METHODS

### 2.1 Plasma sample collection

A total of 16 plasma samples of patients with AML were collected received in Three Gorges Hospital Affiliated to Chongqing University from 2019 to 2020. The patients were pathologically diagnosed as AML. And 16 healthy examiners were collected as normal control group. A total of 3 mL venous blood was collected with EDTA anticoagulation tubes to centrifuge at 1000 g for 10 min to took upper plasma. EDTA‐K2 was collected anticoagulated plasma of all participants to put into EP tubes without RNase and DNase, and frozen in −80°C refrigerator for further use. All experiments have got approval from the Ethics Committee of Three Gorges Hospital Affiliated to Chongqing University Approval No. 20180825669, patients and their families have been notified to collect specimens and sign informed consent.

### 2.2 Cell culture

Normal HS‐5 cells and AML cell lines (HL‐60, NB4, and Thp‐1) purchased from ATCC were cultured in RPMI 1640 medium (Gibco) containing 10% FBS, 100 U/mL penicillin, and 0.1 mg/mL streptomycin, placed in constant temperature incubators at 37°C, with 5% CO_2_ and 95% humidity. Applied cells in logarithmic growth phase for our experiment. The cell lines used in the study could not represented all AML status.

### 2.3 Cell transfection

After transfected mimic NC, miR‐1294 mimic, inhibitor NC, miR‐1294 inhibitor (100 nM), pcDNA3.1‐circFN1, si‐circFN1, and si‐ARHGEF10L (Jima Gene, China) in HL‐60 cells (2.5 ug each), all transfection operations were carried out strictly based on lipofectamine 2000 transfection instructions through transfection kit (Thermo). The successfully transfected cells were cultured in DMEM serum‐free medium and constant 37°C temperature incubator with 5% CO_2_.

### 2.4 Real‐time quantitative PCR experiment

TRIzol Reagent (Life Technologies, USA) was used to extract total RNA. The concentration and purity of RNA were detected with an ultra‐micro spectrophotometer NanoDrop2000 (Thermo), reverse transcription on total RNA was performed via Revert Aid First Strand cDNA Synthesis Kit (Thermo), cDNA template was synthesized by using PrimeScriptTM RT Master Mix Kit (TaKaRa, Japan) and reversed transcription reaction via poly‐A method. The qPCR was performed on the StepOne™ Real‐Time PCR System (Applied Biosystems) with a SYBR Premix Ex Taq Kit (TaKaRa, Japan) then performed through fluorescent RT‐qPCR analyzer (CFX Connect, USA). β‐actin was used as the internal control to standardize RNA expression. Human 18SrRNA was amplified as an endogenous control for circFN1. Human U6 RNA was amplified as an endogenous control for miR‐1294. All the reactions were performed in triplicate. According to the manufacturer's instructions, the PCRs were conducted at 95°C for 30 s, followed by 40 cycles of 95°C for 3 s, and 60°C for 30 s in the StepOne™ Real‐Time PCR System (Applied Biosystems). Data analysis was performed through 2‐ΔΔCt,^26^ and the formula was in the following:

$$\text{ΔΔCt}={\left[{\mathrm{Ct}}_{\text{(target gene)}}–{\mathrm{Ct}}_{\text{(internal reference gene)}}\right]}_{\text{experimental group}}–{\left[{\mathrm{Ct}}_{\text{(target gene)}}–{\mathrm{Ct}}_{\text{(internal reference gene)}}\right]}_{\text{control group}}.$$

See Table 1 for the amplified primer sequences of each gene and its primers.

**TABLE 1:** Primer sequence.

Name of primer	Sequences
circFN1‐F	GGAGAAGTATGTGCATGGTGTCA
circFN1‐R	TGCAGATTTCCTCGTGGGTTG
miR‐1294‐F	TGTGAGGTTGGCATTGTTGTCTGT
miR‐1294‐R	GTGCAGGGTCCGAGGTATTC
ARHGEF10L‐F	AGTGCCAGGTGGTGTTCTTC
ARHGEF10L‐R	AAGAGGTCCCCGATCTTCTC
U6‐F	CTCGCTTCGGCAG CACA
U6‐R	AACGCTTCACGAATTTGCGT
β‐actin‐F	CTCCATCCTGGCCTCGCTGT
β‐actin‐R	GCTGTCACCTTCACCGTTCC
18S rRNA‐F	GGAGTATGGTTGCAAAGCTGA
18S rRNA‐R	ATCTGTCAATCCTGTCCGTGT

### 2.5 Western blotting

The cells were rinsed gently with pre‐cooled PBS buffer for three times. Then added 150–250 μL of lysis solution (Solebo, China) in accordance with the number of cells per well of the 6‐well plates, of which each 1 mL RIPA was added to 10 μL PMSF to make the final PMSF concentration at 1 mM. Centrifuged the lysed samples at 12,000 g for 5 min to took the supernatant, performed BCA protein quantification kit (Norwegian, China) to detect protein concentration of each sample, separated the protein extract (30 μg per channel) on 10% SDS‐PAGE, transferred it to PVDF membrane (Millipore, MA), and later blocked it with 5% skim milk at room temperature for 1 h. After that, the membrane was placed into the incubators with rabbit anti‐human ARHGEF10L (ab121866, 1:1000, Abcam, USA), overnight at 4°C. later washed 3 times with TBST, 10 min each, with horseradish peroxidase‐labeled goat anti‐rabbit IgG (1:5000, Solebo), incubated for 1 h at room temperature, and washed 3 times with TBST, 10 times each. Chemiluminescence imaging system (Shanghai Tianneng Technology Co., Ltd.) was applied for visualization and data analysis, β‐actin (4970S, 1:1000, Cell Signaling Technology, USA) as the loading control. The experiment was carried out three times independently.

### 2.6 CCK‐8 for cell proliferation detection

Cells were seeded in each group into a 96‐well plate 24 h after transfection. 100 μL of diluted cell suspension (1 × 10^5^ cells/ml) was added in each well, and 3 replicates were set for each sample. After incubating them in incubators for 24, 48, 72, and 96 h respectively, 10 μL of CCK‐8 reagent (Dojindo, Japan) was added to each well. After incubation for 2 h in incubators, the absorbance was measured at 450 nm.

### 2.7 Transwell experiment

Cell migration and invasion experiments were proceeded in 8 μm‐pore‐size polyethylene terephthalate (PET)‐based migration chamber and BD Matrigel gel invasion chamber. A serum‐free cell suspension was prepared to perform cell count, and 2 × 10^4^ cells were taken to put in 0.6 mL sterile EP tubes. DMEM was added up to 200 μL. Later 800 μL of DMEM containing 15% FBS was added to the 24‐well plates, the Transwell chamber was placed on top, and gently 200 μL of cell suspension was added to the upper chamber to make the cells evenly distributed in the wells. After incubating them for 48 h, the Transwell cell was taken out, the culture medium was discarded in wells, washed twice with PBS, fixed with 100% methanol for 10 min, then discarded the methanol. An appropriate amount of Giemsa staining solution was added to stain for over 40 min, and gently washed off the staining solution in water, and non‐migrated cells were gently wiped off in upper layer with cotton swabs. After natural drying, inverted microscopes (Zeiss Axioskop 2) was applied to count at 200 times magnification. AML cell migration and invasion ability was calculated in reference to the average number of cells in all regions, and expressed as the relative ratio to the control cells. The experiment was carried out three times independently.

### 2.8 Terminal deoxynucleotidyl transferase‐mediated dUTP‐biotin nick end labeling (TUNEL)

TUNEL Apoptosis Detection Kit was applied for cell apoptosis detection according to the manufacturer's manual (Thermo Fisher Scientific, Waltham, MA, USA). Confocal microscopy (Olympus BX51TRF; Olympus Corporation) (magnification, ×100) was applied to scan the signal from three random fields and count the number of TUNEL positive cells. The apoptosis index was used to measure the degree of apoptosis.

### 2.9 Flow cytometry

Flow cytometry was used for cell apoptosis detection. Centrifuge sample and aspirate supernatant. Resuspend cell pellets (10^6^ cell each tube) 0.5 mL of Hanks balanced salt solution (HBSS), then add 1.5 mL of ethanol. Incubate for 1 h at 4°C. Centrifuge samples at 800 g for 10 min and aspirate supernatant. Add to the pellet 250 μL HBSS, 250 μL RNAse and 500 μL propidium iodide. Incubate the mixture for 15 min at room temperature and then maintain it at 4°C in the dark until flow cytometric analysis. Each experiment was repeated three times.

### 2.10 Dual luciferase reporter gene experiment

Online prediction software Starbase (http://starbase.sysu.edu.cn/) was used to predict the binding target sites of circFN1 and miR‐1294, as well as via RNA22 (https://cm.jefferson.edu/rna22/), PicTar (https://pictar.mdc-berlin.de/), PITA (https://genie.weizmann.ac.il/pubs/mir07/mir07_data.html), microT (http://www.microrna.gr/microT-CDS) online prediction software to predicted the binding target sites of miR‐1294 and ARHGEF10L, in accordance with the prediction findings, MUT and WT sequences of the binding sites of circFN1 and miR‐1294, miR‐1294 and ARHGEF10L were respectively designed. MUT and WT sequence fragments were cloned, combining them with pGL3‐Basic vector (Promega), and later respectively miR‐1294 mimic or miR‐1294 inhibitor or corresponding NC were transfected into 293T cells, and luciferase reporter gene kit (purchased from Beijing Yuanpinghao Biotechnology Co., Ltd.) was applied to detect luciferase activity in cells of each group 48 h after transfection.

### 2.11 Statistical analysis

Statistical analysis was performed with GraphPad Prism 8 software. All data expressed as mean ± standard deviation ($\bar{x}$ ± s), via *t* test for the comparison between two groups, one‐way analysis of variance (ANOVA) for multiple comparison among groups, and Dunnett's multiple comparisons test for subsequent comparisons. *P* < 0.05 mean statistical significance.

## 3 RESULTS

### 3.1 circFN1 promoted AML cell proliferation, migration, and invasion

**FIGURE 1:**
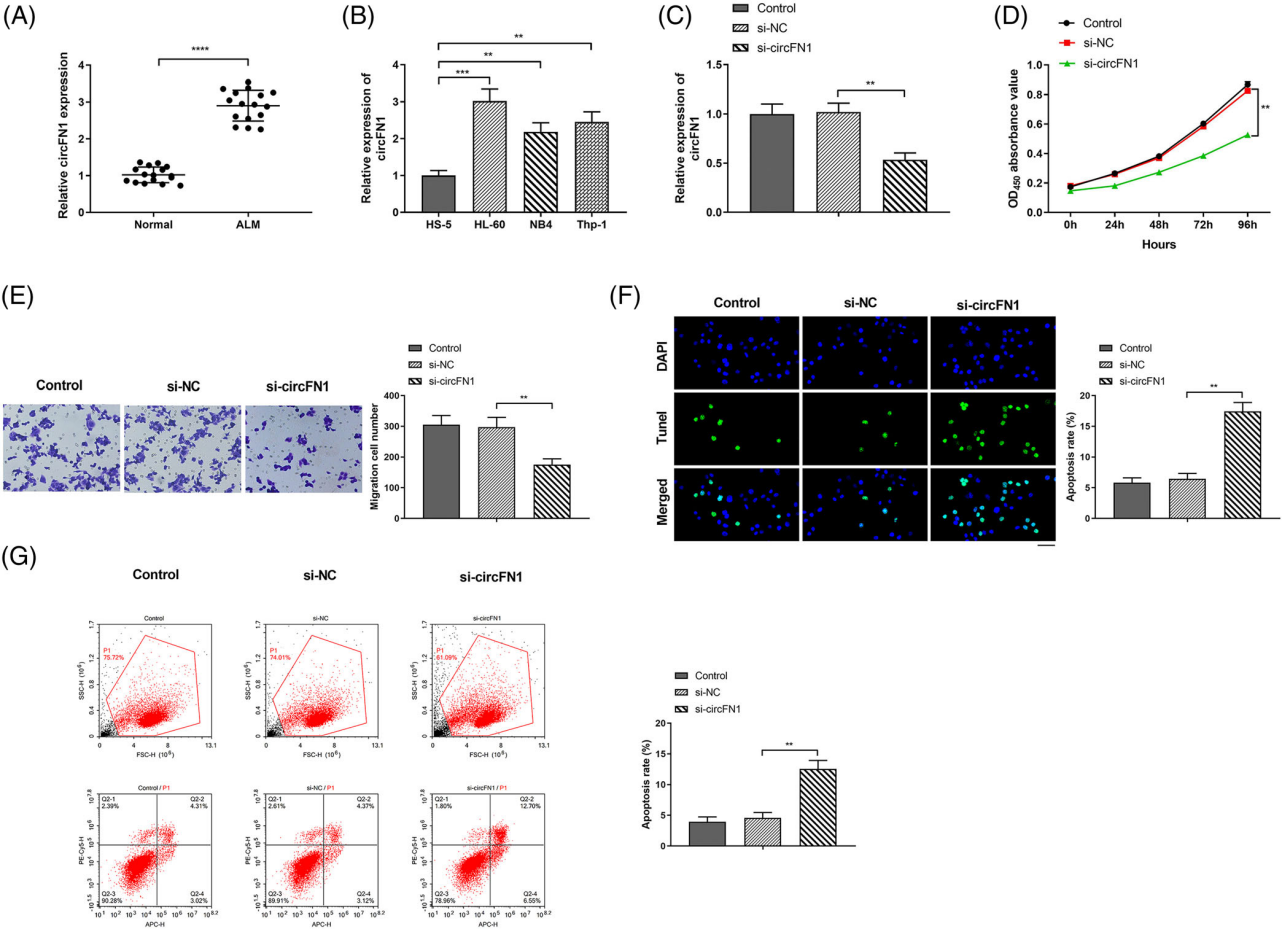
circFN1 promoted acute myeloid leukemia (AML) cell proliferation and invasion, while repressed apoptosis. (A) RT‐qPCR to detect circFN1 expression level in the plasma of each group; (B) RT‐qPCR to test circFN1 expression level in cells of each group; (C) RT‐qPCR to detect circFN1 expression level in HL‐60 cells of each group; (D) CCK‐8 experiment to detect HL‐60 cell proliferation ability of each group; (E) Transwell experiment to detect HL‐60 cell invasion ability of each group; (F) TUNEL staining to detect cell apoptosis; Scale bar: 40 μm; (G) Flow cytometry to detect cell apoptosis. *P < 0.05, **P < 0.01, ***P < 0.001, ****P < 0.0001.

hsa_circ_0058124 (located at chr2:216270960–216274462 and derived from gene FN1 exons 15–19, with a spliced mature sequence length of 864 bp) has been termed as “circFN1” (http://www.circbase.org/cgi‐bin/singlerecord.cgi?id=hsa_circ_0058124; http://circnet.mbc.nctu.edu.tw/, by searching circFN1). CircFN1, highly expressed in gastric cancer cells, has impact of inhibiting cell apoptosis and increasing cell resistance.^20^ To ascertain the impact of circFN1 on AML, clinical samples were collected and performed via RT‐qPCR experiments. We found that circFN1 was highly expressed in plasma in AML group (Figure 1A, *P* < 0.0001) in comparison with the Normal group. After detecting circFN1 expression level in human bone marrow stromal HS‐5 cells and three AML cell lines (HL‐60, NB4, and Thp‐1), we found that in comparison with HS‐5 cells, circFN1 was up‐regulated in AML cell lines (Figure 1B, *P* < 0.01, *P* < 0.001), and HL‐60 was obviously proliferated. Therefore, we speculate that circFN1 promotes AML cell proliferation, migration, and invasion. Based on RT‐qPCR, circFN1 mRNA content in si‐circFN1 group was signally reduced in contrast to si‐NC group (Figure 1C, *P* < 0.01), indicating that circFN1 was successfully silenced. CCK‐8 experiment showed in contrast to si‐NC group, cell proliferation ability in si‐circFN1 group was visually reduced (Figure 1D, *P* < 0.01), indicating that circFN1 promoted cell proliferation. Based on Transwell experiment, we found that in comparison with si‐NC group, the cell invasion ability in si‐circFN1 group was signally reduced (Figure 1E, *P* < 0.01), indicating that circFN1 promoted cell invasion. TUNEL assay and Flow cytometry detection showed the apoptosis ability of si‐circFN1 group was apparently enhanced versus si‐NC group (Figure 1F, G, *P* < 0.01), indicating that circFN1 repressed apoptosis. There was no significant difference between control group and si‐NC group in each detection index. In summary, circFN1 promotes AML cell proliferation and invasion but represses apoptosis.

### 3.2 circFN1 could negatively regulated miR‐1294

**FIGURE 2:**
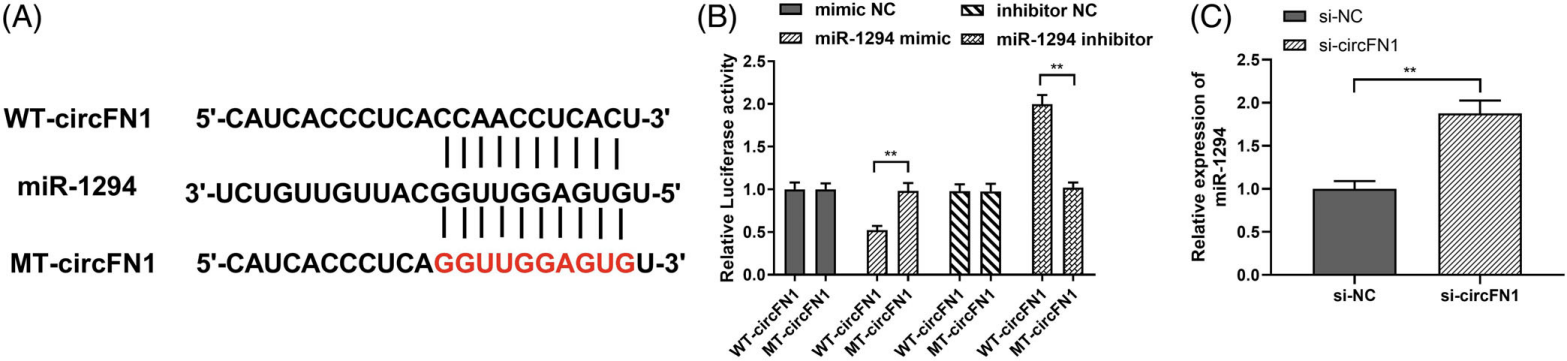
circFN1 negatively regulated miR‐1294. (A) Biology online software Starbase to predict the design of the targeting sites of circFN1 and miR‐1294 as well as the mutation sites of LINC00852; (B) Dual luciferase reporter gene experiment to calculate the relative activity of each plasmid luciferase; (C) RT‐qPCR to detect miR‐1294 expression level in HL‐60 cells of each group. **P < 0.01.

circRNA can act as a sponge of miRNAs to regulate gene expression in many cancers, thereby exerting biological functions.^13^ In order to probe into the mechanism, we applied Starbase online database (http://starbase.sysu.edu.cn/index.php) to predict circFN1 target gene, finding that circFN1 and miR‐1294 had direct binding sites. Therefore, we speculate that circFN1 negatively regulates miR‐1294 by targeting to promote AML cell proliferation, migration, and invasion. Through designing the mutation sites in circFN13′‐UTR region and miR‐1294 (Figure 2A) to conduct dual luciferase reporter gene experiment, we found that luciferase activity in miR‐1294 mimic + MT‐circFN1 group was signally elevated (*P* < 0.01) in comparison with miR‐1294 mimic + WT‐circFN1 group; in contrast to miR‐1294 inhibitor + WT‐circFN1 group, luciferase activity in miR‐1294 inhibitor + MT‐circFN1 group was significantly decreased (*P* < 0.01) (Figure 2B). This indicates that miR‐1294 and circFN1 directly bind to each other. RT‐qPCR revealed that compared to si‐NC group, miR‐1294 expression in si‐circFN1 group was significantly elevated (Figure 2C, *P* < 0.01). These indicate that circFN1 negatively regulates miR‐1294 through targeting.

### 3.3 miR‐1294 inhibited AML cell proliferation, migration, and invasion

**FIGURE 3:**
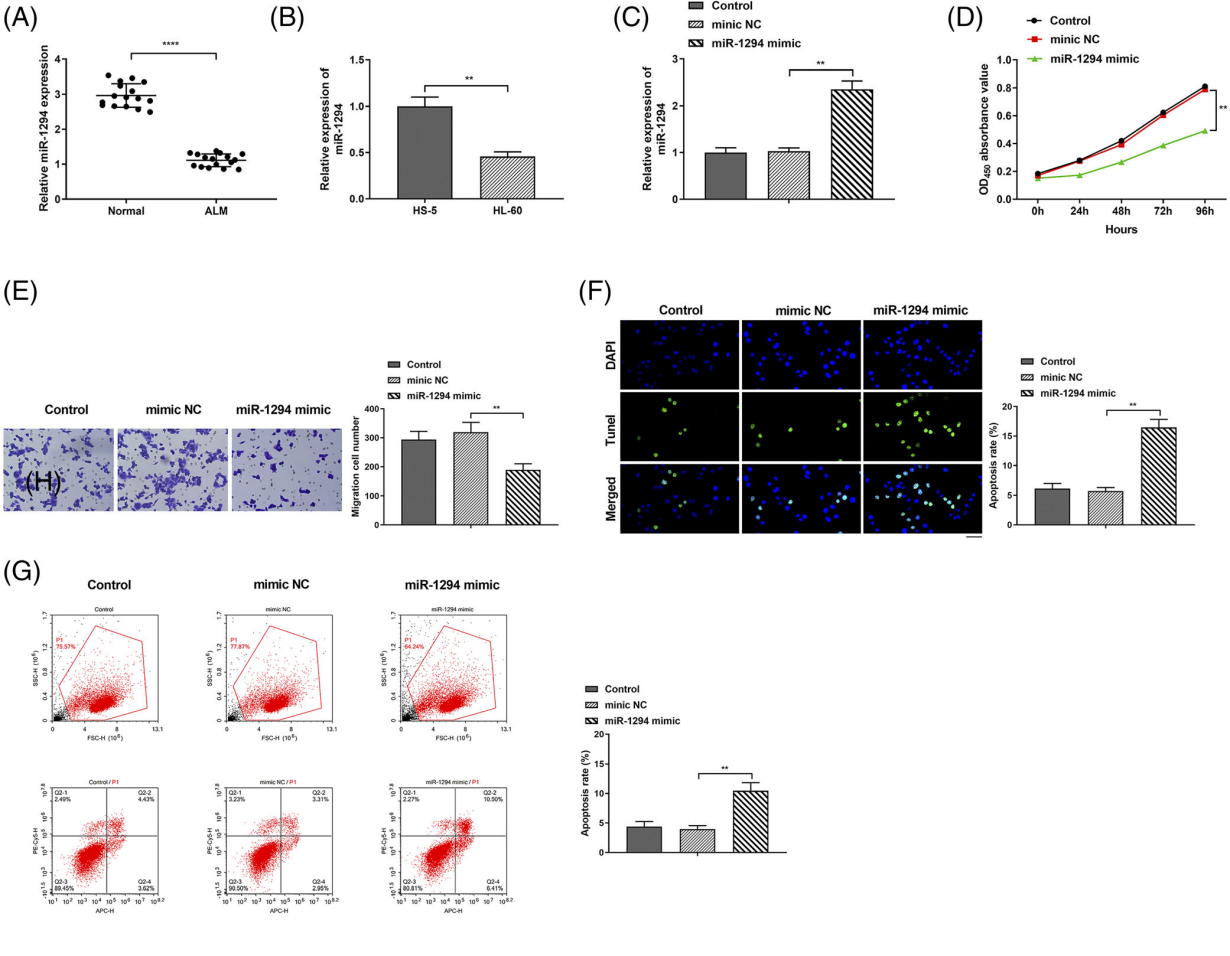
miR‐1294 inhibited acute myeloid leukemia (AML) cell proliferation, migration and invasion. (A) RT‐qPCR to detect miR‐1294 expression level in plasma of each group; (B) RT‐qPCR to examine miR‐1294 expression level in cells of each group; (C) RT‐qPCR to detect miR‐1294 expression level in HL‐60 cells of each group; (D) CCK‐8 experiment to test HL‐60 cell proliferation ability in each group; (E) Transwell experiment to detect HL‐60 cell invasion ability in each group; (F) TUNEL staining to detect cell apoptosis. Scale bar: 40 μm; (G) Flow cytometry to detect cell apoptosis. **P < 0.01, ****P < 0.0001.

miR‐1294 can inhibit cancer cell growth in Esophageal Squamous Cell Carcinoma and liver cancer.^22^, ^23^, ^24^ We speculate that miR‐1294 is available to inhibit AML cell proliferation, migration, and invasion. RT‐qPCR experiment found that miR‐1294 was down‐expressed in AML patients (Figure 3A, *P* < 0.0001); in comparison with HS‐5 group, miR‐1294 expression in HL‐60 group was signally reduced (Figure 3B, *P* < 0.01), indicating that miR‐1294 was under‐expressed in AML cells. miR‐1294 expression in miR‐1294 mimic group was obviously higher than that in mimic NC group (Figure 3C, *P* < 0.01), indicating that miR‐1294 was successfully over‐expressed. CCK‐8 experiment revealed that compared to mimic NC group, the cell proliferation ability in miR‐1294 mimic group was signally decreased (Figure 3D, *P* < 0.01), indicating that miR‐1294 inhibited cell proliferation. Transwell experiment revealed that cell invasion ability in miR‐1294 mimic group was signally reduced in contrast to mimic NC group (Figure 3E, *P* < 0.01), indicating that miR‐1294 inhibited cell invasion. Data of TUNEL staining and flow cytometry implied that in contrast to mimic NC group, the miR‐1294 mimic group had visually elevated cell apoptosis ability (Figure 3F, G, *P* < 0.01), indicating that miR‐1294 accelerated cell apoptosis. The control group and mimic NC group showed no obvious differences in detection indicators. In summary, miR‐1294 inhibits AML cell proliferation and invasion but accelerates apoptosis.

### 3.4 miR‐1294 negatively regulated ARHGEF10L through targeting

**FIGURE 4:**
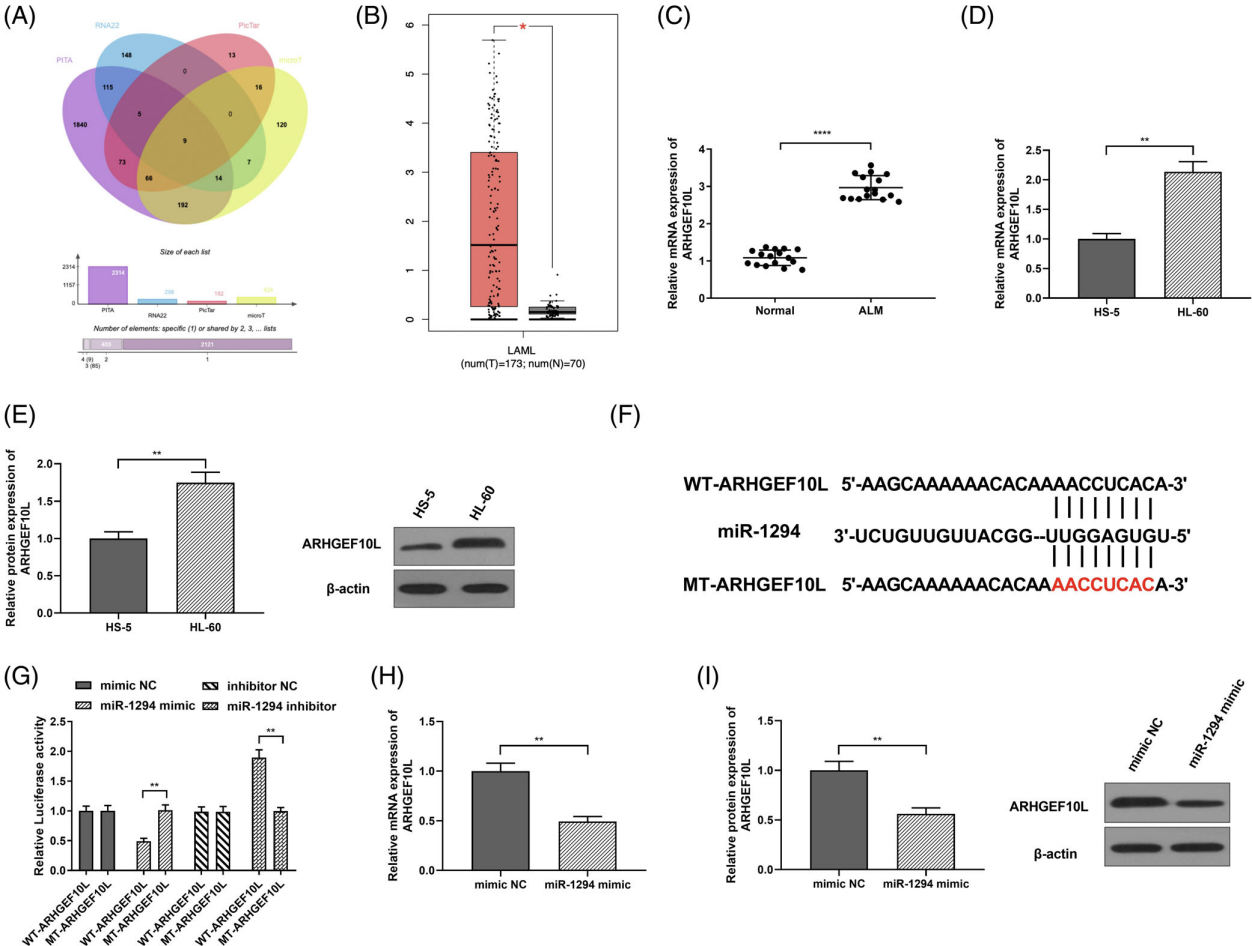
miR‐1294 negatively regulated ARHGEF10L. (A) Online software RNA22, PicTar, PITA, and microT to predict miR‐1294 downstream mRNA, combined with Venn diagram analysis; (B) GEPIA2 online database analysis to predict gene expression in acute myeloid leukemia (AML); (C) RT‐qPCR to detect ARHGEF10L mRNA expression level in plasma of each group; (D) RT‐qPCR to examine ARHGEF10L mRNA expression level in cells of each group; (E) Western blot to detect ARHGEF10L protein expression level in cells of each group; (F) Designing the mutation sites in ARHGEF10L 3′‐UTR region and miR‐1294; (G) Conducting dual luciferase reporter gene experiment to calculate each plasmid luciferase relative activity; (H) RT‐qPCR to detect ARHGEF10L mRNA expression level in HL‐60 cells of each group; (I) Western blot to detect ARHGEF10L protein expression level in HL‐60 cells. *P < 0.05, **P < 0.01, ****P < 0.0001.

We then used RNA22, PicTar, PITA, and microT online prediction software to predict the downstream mRNA of miR‐1294. Through Venn diagram analysis, we concluded that miR‐1294 predicted by RNA22, PicTar, PITA, and microT had 9 common target genes: DDI2, ARHGEF10L, KCTD21, ASIC1, ARHGAP28, ONECUT2, CLASP1, SULF2, and ROBO1 (Figure 4A). Using GEPIA2 online database (http://gepia2.cancer-pku.cn/), but we found only ARHGEF10L was highly expressed in AML (Figure 4B, *P* < 0.05), which has not been reported in researches on AML. After RT‐qPCR experiment, we found that ARHGEF10L was highly expressed in AML patients (Figure 4C, *P* < 0.0001). Based on further research, in comparison with HS‐5 group, ARHGEF10L mRNA and protein expression in HL‐60 group were signally elevated (Figure 4D, E, *P* < 0.01). Therefore, our work designed the mutation sites in ARHGEF10L3′‐UTR region and miR‐1294 (Figure 4F), as well as performed dual luciferase reporter gene experiment. It was found that in comparison with miR‐1294 mimic + WT‐ARHGEF10L group, luciferase activity of miR‐1294 mimic + MT‐ARHGEF10L group was significantly reduced (*P* < 0.01); in contrast to miR‐1294 inhibitor + WT‐ARHGEF10L group, luciferase activity of miR‐1294 inhibitor + WT‐ARHGEF10L group was signally elevated (*P* < 0.01) (Figure 4G, *P* < 0.01), indicating that miR‐1294 and ARHGEF10L directly bound to each other. RT‐qPCR and western blot experiments revealed that ARHGEF10L expression in miR‐1294 mimic group was signally reduced in contrast to mimic NC group (Figure 4H, I, *P* < 0.01). These data indicate that miR‐1294 negatively regulates ARHGEF10L.

### 3.5 circFN1 promoted AML cell proliferation, migration, and invasion through miR‐1294/ARHGEF10L axis

**FIGURE 5:**
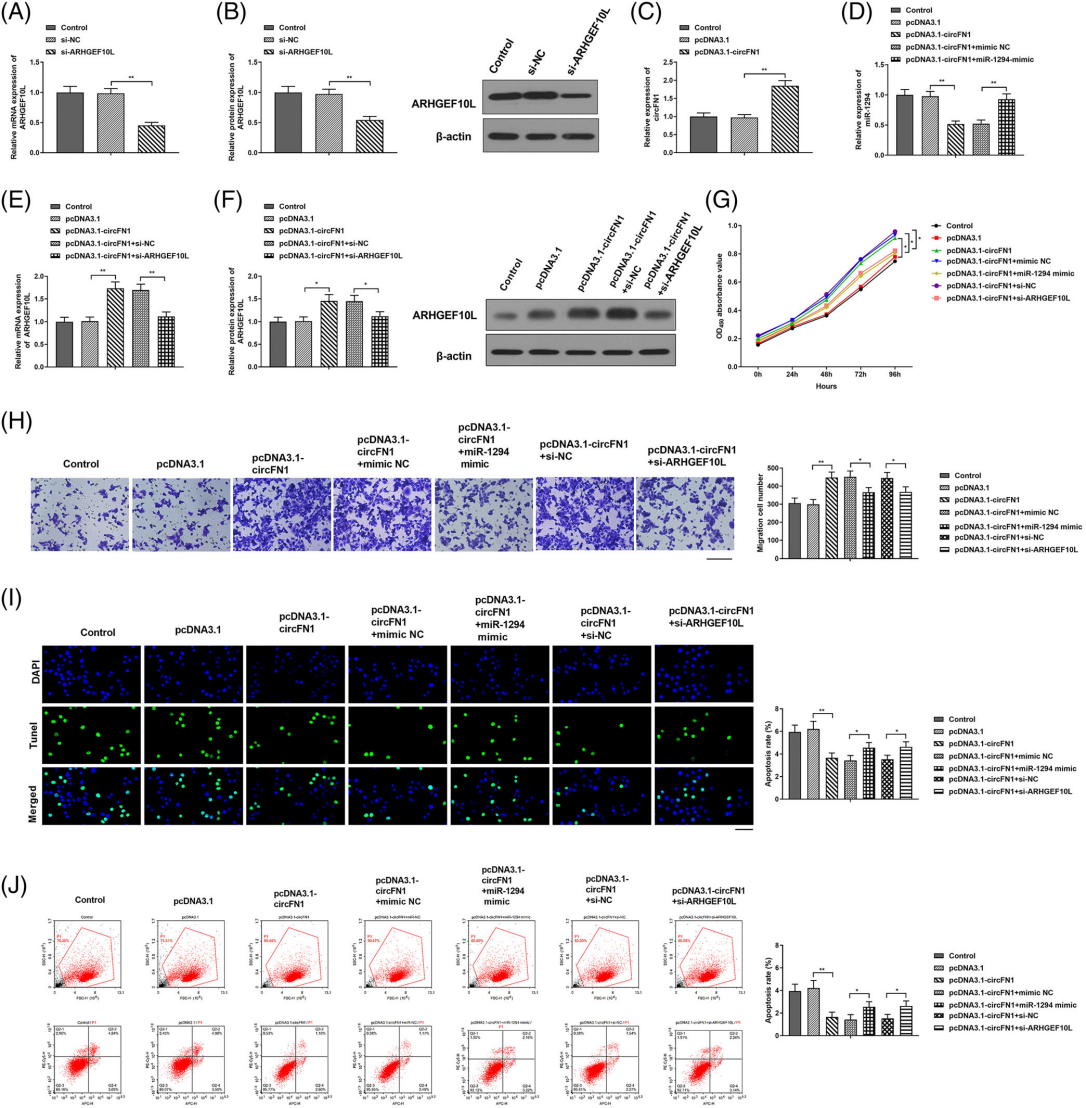
circFN1 promoted acute myeloid leukemia (AML) cell proliferation, migration and invasion via miR‐1294/ARHGEF10L axis. (A) RT‐qPCR to detect ARHGEF10L mRNA expression levels in HL‐60 cells of each group; (B) Western blot to detect ARHGEF10L protein expression level in HL‐60 cells of each group; (C) RT‐qPCR to detect circFN1 expression level in HL‐60 cells of each group; (D) RT‐qPCR to examine miR‐1294 expression level in HL‐60 cells of each group: (E) RT‐qPCR to test ARHGEF10L mRNA expression level in HL‐60 cells of each group; (F) Western blot to detect ARHGEF10L protein expression level in HL‐60 cells of each group; (G) CCK‐8 experiment to examine HL‐60 cell proliferation ability of each group; (H) Transwell experiment to test HL‐60 cell invasion ability of each group; (I) TUNEL staining to detect cell apoptosis, Scale bar: 40 μm; (G) Flow cytometry to detect cell apoptosis. *P < 0.05, **P < 0.01.

circFN1 is available to promote AML cell proliferation and invasion, but repress apoptosis through inhibiting miR‐1294. miR‐1294 inhibited expression of ARHGEF10L. In liver cancer, ARHGEF10L is available to promote tumorigenesis.^25^ Our work found that in contrast to si‐NC group, ARHGEF10L expression in si‐ARHGEF10L group was signally reduced (Figure 5A, B, *P* < 0.01), indicating that ARHGEF10L was successfully silenced. RT‐qPCR detection revealed that circFN1 expression in pcDNA3.1‐circFN1 group was significantly higher than that in pcDNA3.1 group, indicating that circFN1 was successfully over‐expressed (Figure 5C, *P* < 0.01); in comparison with pcDNA3.1‐circFN1 + mimic NC group, miR‐1294 content in pcDNA3.1‐circFN1 + miR‐1294‐mimic group was visually increased (Figure 5D, *P* < 0.01); in contrast to pcDNA3.1‐circFN1 + si‐NC group, ARHGEF10L content in pcDNA3.1‐circFN1 + si‐ARHGEF10L group was significantly reduced (Figure 5E, *P* < 0.01). Western blot experiment showed that in comparison with pcDNA3.1‐circFN1 + si‐NC group, ARHGEF10L content in pcDNA3.1‐circFN1 + si‐ARHGEF10L group was obviously decreased (Figure 5F, *P* < 0.05). In contrast to pcDNA3.1 group, pcDNA3.1‐circFN1 group had signally higher cell proliferation and migration capabilities, and apparently reduced apoptosis capability; in contrast to pcDNA3.1‐circFN1 + mimic NC group, proliferation and migration capabilities of pcDNA3.1‐circFN1 + miR‐1294‐mimic group cell were significantly reduced, but cell apoptosis capability was clearly enhanced; in comparison with pcDNA3.1‐circFN1 + si‐NC group, cell proliferation and migration capabilities of pcDNA3.1‐circFN1 + si‐ARHGEF10L group were signally reduced, but cell apoptosis capability was clearly enhanced (Figure 5G–J, *P* < 0.05, *P* < 0.01), indicating that over‐expressing miR‐1294 or silencing ARHGEF10L can inhibit the promotion role of circFN1 in AML cell proliferation and invasion, as well as the repression in cell apoptosis. There was no obvious difference in detection indexes between Control group and pcDNA3.1 group. CircFN1 can negatively regulate miR‐1294 through targeting with ARHGEF10L acting as the target of miR‐1294, which reveals that circFN1 promotes AML cell proliferation and invasion, and refrains cell apoptosis via miR‐1294/ARHGEF10L axis.

## 4 DISCUSSION

AML is a malignant tumor with short survival and poor prognosis.^27^ It is necessary to probe into new and effective AML diagnostic and prognostic indicators. Increasing evidences describe circRNA dysregulation in various cancers,^28^ and the role of circRNA in AML has been continuously elucidated. CircFN1, highly expressed in gastric cancer cells, can inhibit cell apoptosis and increasing cell resistance.^20^ Here, in accordance with the GEPIA online database and clinical sample analysis, it was found that circFN1 in plasma samples of AML patients was signally up‐regulated in comparison with healthy controls, indicating that circ‐PTK2 may be an oncogene in AML. Through silencing circFN1, we found that circFN1 promoted AML cell proliferation and invasion, but refrained apoptosis.

CircRNA participates in human cancer occurrence via its miRNA sponge activity.^29^ For example, circ_0072995 accelerates breast cancer cell movement through acting as a sponge for miR‐30c‐2‐3p.^30^ In esophageal squamous cell carcinoma and liver cancer, miR‐1294 can inhibit cancer cell growth.^22^, ^23^, ^24^ We verified through bioinformatics analysis and dual luciferase experiment that miR‐1294 could be a direct target of circ FN1. Through silencing circFN1, our work found that miR‐1294 is negatively regulated by circFN1 in AML cells. CircFN1 promotes AML cell proliferation and invasion, but refrains apoptosis through down‐regulating miR‐1294. At present, studies have found that MiRNA‐mRNA regulation axis is involved in regulating cell functions. For example, miR‐214 inhibits liver cancer cell proliferation and migration through reducing FOXM1 levels.^31^ miR‐370 exerts a tumor suppressor impact through targeting FOXM1 in AML cells.^32^ The direct interaction between miR‐1294 and ARHGEF10L has been verified via dual luciferase reporter experiment. Based on our experiments, miR‐1294 exerts biological functions through targeting ARHGEF10L, and circFN1 releases ARHGEF10L through chelating miR‐1294, thereby up‐regulating ARHGEF10L level in AML cells. ARHGEF10L, belonging to guanine nucleotide exchange factor family, is available to induce GDP/GTP exchange on RhoA,^33^ and increase in RhoA/Rho kinase signal conduction to promote cancer development and metastasis.^34^, ^35^, ^36^, ^37^ ARHGEF10L acts through RhoA‐ROCK1‐ERM signal conduction (an essential pathway in tumorigenesis) and stimulates EMT expression in liver and gastric cancer cells.^25^, ^38^ Abnormal ROR1 expression appears in many malignant tumors and is related to Rho‐GTPase activation; high ROR1 expression level in leukemia cells is related to poor prognosis.^39^ Abnormal ARHGEF10L expression appears in various malignant tumors and is also related to Rho‐GTPase activation. Therefore, we reasonably speculate that ARHGEF10L may be involved in various cancer occurrences through RhoA/Rho kinase signaling pathway.

## 5 CONCLUSION

To sum up, we clarified the carcinogenic impact of circFN1 in AML. CircFN1 promotes AML cell proliferation and invasion, but restrains apoptosis. Additionally, through bioinformatics analysis, miR‐1294 and ARHGEF10L were found as circFN1 downstream genes. CircFN1 up‐regulates ARHGEF10L level through its miR‐1294 sponge activity, thereby promoting AML cell proliferation and invasion, and restraining apoptosis.

## CONFLICT OF INTEREST STATEMENT

The authors declare no conflicts of interest.

## References

[1] Wang X , Jin P , Zhang Y , Wang K . CircSPI1 acts as an oncogene in acute myeloid leukemia through antagonizing SPI1 and interacting with microRNAs. Cell Death Dis. 2021;12(4):297. 33741901 10.1038/s41419-021-03566-2 PMC7979773

[2] Döhner H . Implication of the molecular characterization of acute myeloid leukemia. Hematol Am Soc Hematol Educ Program. 2007;412‐9:412–419. 10.1182/asheducation-2007.1.412 18024659

[3] Mrózek K , Marcucci G , Paschka P , Whitman SP , Bloomfield CD . Clinical relevance of mutations and gene‐expression changes in adult acute myeloid leukemia with normal cytogenetics: are we ready for a prognostically prioritized molecular classification? Blood. 2007;109(2):431–448. 16960150 10.1182/blood-2006-06-001149 PMC1785102

[4] Bennett JM , Catovsky D , Daniel MT , Flandrin G , Galton DA , Gralnick HR , et al. Proposed revised criteria for the classification of acute myeloid leukemia. A report of the French‐American‐British cooperative group. Ann Intern Med. 1985;103(4):620–625. 3862359 10.7326/0003-4819-103-4-620

[5] Vardiman JW , Thiele J , Arber DA , Brunning RD , Borowitz MJ , Porwit A , et al. The 2008 revision of the World Health Organization (WHO) classification of myeloid neoplasms and acute leukemia: rationale and important changes. Blood. 2009;114(5):937–951. 19357394 10.1182/blood-2009-03-209262

[6] Kuykendall A , Duployez N , Boissel N , Lancet JE , Welch JS . Acute myeloid leukemia: the good, the bad, and the ugly. Am Soc Clin Oncol Educ Book. 2018;38:555–573. 30231330 10.1200/EDBK_199519

[7] Rodrigues ACBDC , Costa RGA , Silva SLR , Dias IRSB , Dias RB , Bezerra DP . Cell signaling pathways as molecular targets to eliminate AML stem cells. Crit Rev Oncol Hematol. 2021;160:103277. 33716201 10.1016/j.critrevonc.2021.103277

[8] Li F , Sutherland MK , Yu C , Walter RB , Westendorf L , Valliere‐Douglass J , et al. Characterization of SGN‐CD123A, a potent CD123‐directed antibody‐drug conjugate for acute myeloid leukemia. Mol Cancer Ther. 2018;17(2):554–564. 29142066 10.1158/1535-7163.MCT-17-0742

[9] Tang X , Ren H , Guo M , Qian J , Yang Y , Gu C . Review on circular RNAs and new insights into their roles in cancer. Comput Struct Biotechnol J. 2021;19:910–928. 33598105 10.1016/j.csbj.2021.01.018 PMC7851342

[10] Abbaszadeh‐Goudarzi K , Radbakhsh S , Pourhanifeh MH , Khanbabaei H , Davoodvandi A , Fathizadeh H , et al. Circular RNA and diabetes: epigenetic regulator with diagnostic role. Curr Mol Med. 2020;20(7):516–526. 31995005 10.2174/1566524020666200129142106

[11] Shabaninejad Z , Vafadar A , Movahedpour A , Ghasemi Y , Namdar A , Fathizadeh H , et al. Circular RNAs in cancer: new insights into functions and implications in ovarian cancer. J Ovarian Res. 2019;12(1):84. 31481095 10.1186/s13048-019-0558-5 PMC6724287

[12] Memczak S , Jens M , Elefsinioti A , Torti F , Krueger J , Rybak A , et al. Circular RNAs are a large class of animal RNAs with regulatory potency. Nature. 2013;495(7441):333–338. 23446348 10.1038/nature11928

[13] Wang F , Nazarali AJ , Ji S . Circular RNAs as potential biomarkers for cancer diagnosis and therapy. Am J Cancer Res. 2016;6(6):1167–1176. 27429839 PMC4937728

[14] Hallajzadeh J , Amirani E , Mirzaei H , Shafabakhsh R , Mirhashemi SM , Sharifi M , et al. Circular RNAs: new genetic tools in melanoma. Biomark Med. 2020;14(7):563–571. 32462914 10.2217/bmm-2019-0567

[15] Jeck WR , Sorrentino JA , Wang K , Slevin MK , Burd CE , Liu J , et al. Circular RNAs are abundant, conserved, and associated with ALU repeats. RNA. 2013;19(2):141–157. 23249747 10.1261/rna.035667.112 PMC3543092

[16] Buratin A , Paganin M , Gaffo E , Dal Molin A , Roels J , Germano G , et al. Large‐scale circular RNA deregulation in T‐ALL: unlocking unique ectopic expression of molecular subtypes. Blood Adv. 2020;4(23):5902–5914. 33259601 10.1182/bloodadvances.2020002337 PMC7724907

[17] Guarnerio J , Bezzi M , Jeong JC , Paffenholz SV , Berry K , Naldini MM , et al. Oncogenic role of fusion‐circRNAs derived from cancer‐associated chromosomal translocations. Cell. 2016;165(2):289–302. 27040497 10.1016/j.cell.2016.03.020

[18] Hirsch S , Blätte TJ , Grasedieck S , Cocciardi S , Rouhi A , Jongen‐Lavrencic M , et al. Circular RNAs of the nucleophosmin (NPM1) gene in acute myeloid leukemia. Haematologica. 2017;102(12):2039–2047. 28971903 10.3324/haematol.2017.172866 PMC5709103

[19] Lux S , Blätte TJ , Gillissen B , Richter A , Cocciardi S , Skambraks S , et al. Deregulated expression of circular RNAs in acute myeloid leukemia. Blood Adv. 2021;5(5):1490–1503. 33683343 10.1182/bloodadvances.2020003230 PMC7948263

[20] Huang XX , Zhang Q , Hu H , Jin Y , Zeng AL , Xia YB , et al. A novel circular RNA circFN1 enhances cisplatin resistance in gastric cancer via sponging miR‐182‐5p. J Cell Biochem. 2020;122:1009–1020. 10.1002/jcb.29641 31898357

[21] Hansen TB , Jensen TI , Clausen BH , Bramsen JB , Finsen B , Damgaard CK , et al. Natural RNA circles function as efficient microRNA sponges. Nature. 2013;495(7441):384–388. 23446346 10.1038/nature11993

[22] Liu K , Li L , Rusidanmu A , Wang Y , Lv X . Down‐regulation of MiR‐1294 is related to dismal prognosis of patients with esophageal squamous cell carcinoma through elevating C‐MYC expression. Cell Physiol Biochem. 2015;36(1):100–110. 25925090 10.1159/000374056

[23] Yang T , Li S , Liu J , Yin D , Yang X , Tang Q , et al. Long non‐coding RNA KRT16P2/miR‐1294/EGFR axis regulates laryngeal squamous cell carcinoma cell aggressiveness. Am J Transl Res. 2020;12(6):2939–2955. 32655821 PMC7344088

[24] Luo Z , Lu L , Tang Q , Wei W , Chen P , Chen Y , et al. CircCAMSAP1 promotes hepatocellular carcinoma progression through miR‐1294/GRAMD1A pathway. J Cell Mol Med. 2021;25(8):3793–3802. 33484498 10.1111/jcmm.16254 PMC8051675

[25] Tang J , Liu C , Xu B , Wang D , Ma Z , Chang X . ARHGEF10L contributes to liver tumorigenesis through RhoA‐ROCK1 signaling and the epithelial‐mesenchymal transition. Exp Cell Res. 2019;374(1):46–68. 30444969 10.1016/j.yexcr.2018.11.007

[26] Burja B , Kuret T , Janko T , Topalović D , Živković L , Mrak‐Poljšak K , et al. Olive leaf extract attenuates inflammatory activation and DNA damage in human arterial endothelial cells. Front Cardiovasc Med. 2019;6:56. 31157238 10.3389/fcvm.2019.00056 PMC6531989

[27] Prada‐Arismendy J , Arroyave JC , Röthlisberger S . Molecular biomarkers in acute myeloid leukemia. Blood Rev. 2017;31(1):63–76. 10.1016/j.blre.2016.08.005 27639498

[28] Lei B , Tian Z , Fan W , Ni B . Circular RNA: a novel biomarker and therapeutic target for human cancers. Int J Med Sci. 2019;16(2):292–301. 30745810 10.7150/ijms.28047 PMC6367529

[29] Panda AC . Circular RNAs act as miRNA sponges. Adv Exp Med Biol. 2018;1087:67–79. 30259358 10.1007/978-981-13-1426-1_6

[30] Zhang HD , Jiang LH , Hou JC , Zhou SY , Zhong SL , Zhu LP , et al. Circular RNA hsa_circ_0072995 promotes breast cancer cell migration and invasion through sponge for miR‐30c‐2‐3p. Epigenomics. 2018;10(9):1229–1242. 30182731 10.2217/epi-2018-0002

[31] Tian C , Wu H , Li C , Tian X , Sun Y , Liu E , et al. Downreguation of FoxM1 by miR‐214 inhibits proliferation and migration in hepatocellular carcinoma. Gene Ther. 2018;25(4):312–319. 29973656 10.1038/s41434-018-0029-4

[32] Zhang X , Zeng J , Zhou M , Li B , Zhang Y , Huang T , et al. The tumor suppressive role of miRNA‐370 by targeting FoxM1 in acute myeloid leukemia. Mol Cancer. 2012;11:56. 22900969 10.1186/1476-4598-11-56 PMC3533721

[33] Winkler S , Mohl M , Wieland T , Lutz S . GrinchGEF—a novel Rho‐specific guanine nucleotide exchange factor. Biochem Biophys Res Commun. 2005;335(4):1280–1286. 16112081 10.1016/j.bbrc.2005.08.025

[34] Chang YW , Bean RR , Jakobi R . Targeting RhoA/rho kinase and p21‐activated kinase signaling to prevent cancer development and progression. Recent Pat Anticancer Drug Discov. 2009;4(2):110–124. 19519534 10.2174/157489209788452830

[35] Chew TW , Liu XJ , Liu L , Spitsbergen JM , Gong Z , Low BC . Crosstalk of Ras and rho: activation of RhoA abates Kras‐induced liver tumorigenesis in transgenic zebrafish models. Oncogene. 2014;33(21):2717–2727. 23812423 10.1038/onc.2013.240

[36] Loirand G . Rho kinases in health and disease: from basic science to translational research. Pharmacol Rev. 2015;67(4):1074–1095. 26419448 10.1124/pr.115.010595

[37] Lazer G , Katzav S . Guanine nucleotide exchange factors for RhoGTPases: good therapeutic targets for cancer therapy? Cell Signal. 2011;23(6):969–979. 21044680 10.1016/j.cellsig.2010.10.022

[38] Tang J , Fang K , Li C , Chang X . ARHGEF10L promotes cervical tumorigenesis via RhoA‐mediated signaling. Evid Based Complement Alternat Med. 2021;2021:6683264. 33833821 10.1155/2021/6683264 PMC8012150

[39] Choi MY , Widhopf GF , Ghia EM , Kidwell RL , Hasan MK , Yu J , et al. Phase I trial: Cirmtuzumab inhibits ROR1 signaling and Stemness signatures in patients with chronic lymphocytic leukemia. Cell Stem Cell. 2018;22(6):951–959.e3. 29859176 10.1016/j.stem.2018.05.018 PMC7001723

